# Calpain‐1 Deficiency Attenuates Atherosclerosis and Improves Cardiac Function Through Vascular–Cardiac Coupling Mechanisms in ApoE‐Deficient Mice

**DOI:** 10.1002/fsn3.72511

**Published:** 2026-11-09

**Authors:** Youming Shi, Havyarimana Juvenal, Yunqi Pan, Shuangyi Li, Wenjiao Gu, Yujie Wei, Ying jiang, Junjie Shao, Sijing Chen, Yunyang Zhao, Ting Liu, Limei Shuai, Shining Zhang, Jixin Liu, Xiaochun Zhou, Futian Tang

**Affiliations:** ^1^ Qinghai Province Cardiovascular and Cerebrovascular Disease Specialist Hospital Xining Qinghai China; ^2^ The Second Hospital & Clinical Medical School Lanzhou University Lanzhou Gansu China; ^3^ Department of Cardiovascular Disease Lanzhou University Second Hospital Lanzhou Gansu China; ^4^ Gansu Province Key Laboratory of Environmental Oncology Lanzhou University Second Hospital Lanzhou Gansu China; ^5^ School of Basic Medical Sciences Lanzhou University Lanzhou Gansu China; ^6^ School of Nuclear and Technology Lanzhou University Lanzhou Gansu China; ^7^ Medical Department of Gansu Provincial People's Hospital Lanzhou Gansu China; ^8^ Department of Nephrology Lanzhou University Second Hospital Lanzhou Gansu China

**Keywords:** ApoE knockout mice, atherosclerosis, calpain‐1, cardiac dysfunction, vascular–cardiac coupling

## Abstract

Calpain‐1 has emerged as an important regulator of vascular remodeling and inflammation; however, its systemic role in atherosclerosis and cardiac dysfunction remains incompletely understood. This study investigated whether genetic deletion of calpain‐1 attenuates atherosclerosis and improves cardiac function in apolipoprotein E‐deficient (ApoE KO) mice. ApoE KO mice and ApoE/calpain‐1 double knockout (A × C DKO) mice were fed a high‐fat diet for 8 weeks, while C57BL/6 mice served as controls. Atherosclerotic lesions, serum lipid profiles, endothelial function, cardiac structure and function, and global proteomic changes were evaluated. A × C DKO mice exhibited significantly reduced atherosclerotic plaque burden and decreased CD68‐positive macrophage infiltration compared with ApoE KO mice. Cardiac magnetic resonance imaging revealed that ApoE KO mice developed ventricular dilation and systolic dysfunction characterized by increased LVESV and LVEDV and reduced LVEF. Calpain‐1 deficiency partially normalized ventricular volumes and significantly improved LVEF, LVSV, and cardiac output. Notably, serum total cholesterol, triglycerides, LDL‐cholesterol, HDL‐cholesterol, and glucose levels were not significantly altered by calpain‐1 deletion, indicating that the protective effects occurred independently of systemic lipid metabolism. Mechanistically, calpain‐1 deficiency suppressed foam‐cell related pathways by downregulating SR‐A and CD36 while restoring cholesterol efflux regulators PPAR‐γ and LXR‐α. Endothelial function was improved as evidenced by increased eNOS activity and NO bioavailability. Aortic DIA proteomic analysis further revealed that calpain‐1 ablation reversed multiple pathogenic pathways including cytoskeletal remodeling, extracellular matrix destabilization, oxidative stress, mitochondrial dysfunction, and endothelial barrier impairment. Collectively, these findings demonstrate that calpain‐1 deficiency attenuates atherosclerotic plaque development and improves cardiac function in ApoE‐deficient mice through lipid‐independent mechanisms involving endothelial protection, reduced foam cell formation, and restoration of vascular homeostasis. Calpain‐1 therefore represents a promising therapeutic target linking vascular pathology with cardiac dysfunction in atherosclerosis.

## Graphical abstract

Calpain‐1 deficiency attenuates atherosclerotic plaque formation and improves cardiac function in ApoE‐deficient mice. These protective effects are associated with reduced macrophage infiltration and foam‐cell formation, improved endothelial function, and restoration of vascular homeostasis without significant changes in systemic lipid profiles.

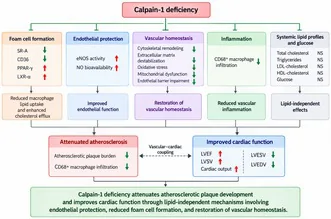

## 1 Introduction

Atherosclerosis is defined as a chronic inflammatory disease of the arterial wall characterized by lipid accumulation, foam cell formation, endothelial dysfunction, and plaque development (Mastrangelo et al. 2025; Zhang, Lin, et al. 2025). Atherosclerosis remains the principal underlying cause of cardiovascular diseases (CVD) such as myocardial infarction and stroke, representing a leading global cause of morbidity and mortality (Li, Gong, et al. 2025; Shekarchizadeh Esfahani et al. 2025; Tang et al. 2025). Despite advances in lipid‐lowering therapies, significant residual cardiovascular risk persists, underscoring the critical need to identify novel therapeutic targets addressing the complex pathophysiology beyond hyperlipidemia, particularly inflammation, endothelial dysfunction, and plaque instability.

Calpains, a family of calcium‐dependent cysteine proteases, have emerged as significant modulators in vascular pathology (Chukai et al. 2025; Niu et al. 2025; Tang et al. 2015; Wen et al. 2025). Calpain‐1, the ubiquitous isoform, is implicated in various cellular processes relevant to atherosclerosis, including cytoskeletal remodeling, cell migration, apoptosis, and signal transduction (Awad et al. 2025; Shi et al. 2025; Taylor et al. 2025). Elevated calpain activity has been documented in human atherosclerotic plaques and experimental models, often correlating with features of plaque vulnerability (Niu et al. 2025; Yin et al. 2017). While prior research has predominantly focused on calpain's role in late‐stage plaque complications, such as smooth muscle cell apoptosis, matrix degradation, and cap thinning, its potential involvement in the initiation and early progression of atherosclerosis, particularly concerning foam cell biology, endothelial health, immune responses, and systemic metabolic regulation within the specific context of hyperlipidemia, remains incompletely defined.

The apolipoprotein E knockout (ApoE KO) mouse is a well‐established and robust model of human atherosclerosis, developing spontaneous hypercholesterolemia and complex atherosclerotic lesions mirroring many features of the human disease (Sanada et al. 2024; Shi et al. 2025). When fed a high‐fat diet (HFD), ApoE KO mice develop accelerated dyslipidemia and atherosclerotic plaque in a short time. This model provides an ideal platform to dissect the specific contributions of calpain‐1 to atherogenesis. Utilizing a genetic approach, we generated ApoE/calpain‐1 double knockout (A × C DKO) mice to directly investigate the consequence of calpain‐1 ablation within the atherogenic milieu driven by ApoE deficiency with additional feeding a HFD (Shi et al. 2025). This model allows for the precise elucidation of calpain‐1's functions without the confounding effects of pharmacological inhibitors.

Importantly, atherosclerosis not only affects vascular structure but also contributes to cardiac dysfunction through vascular–cardiac coupling mechanisms (Li, Luo, et al. 2025). Chronic vascular inflammation, endothelial dysfunction, and arterial stiffening can increase afterload, impair coronary perfusion, and ultimately promote ventricular remodeling and systolic dysfunction (Ceasovschih et al. 2025). Increasing evidence suggests that vascular molecular pathways regulating plaque development may also influence myocardial function through systemic inflammatory, metabolic, and hemodynamic interactions (Gallo and Savoia 2024). Therefore, identifying molecular regulators that simultaneously modulate vascular pathology and cardiac performance may provide new therapeutic strategies for CVD. ApoE deficiency has also been reported to induce myocardial fibrosis, oxidative stress, and progressive cardiac dysfunction, suggesting a close interaction between vascular pathology and myocardial injury (Lo Sasso et al. 2016). Calpain‐1 has been implicated in multiple cardiovascular pathological processes including inflammation, oxidative stress, cytoskeletal remodeling, and endothelial dysfunction (Zhang et al. 2026). Previous studies have shown that increased calpain activity contributes to vascular injury and atherosclerotic plaque progression, whereas inhibition of calpain signaling can alleviate vascular dysfunction (Zhang, Zheng, et al. 2024). However, whether calpain‐1 also participates in the development of cardiac dysfunction associated with atherosclerosis remains poorly understood.

Cardiac magnetic resonance imaging (MRI) is widely regarded as the gold standard for noninvasive evaluation of cardiac structure and function because of its high spatial resolution and excellent soft‐tissue contrast (Chen et al. 2024). Ultra‐high‐field small‐animal MRI systems, such as 9.4 T MRI, provide superior spatial and temporal resolution, enabling accurate and reproducible quantification of ventricular volumes and functional parameters in murine models. Recent studies have demonstrated that 9.4 T cardiac MRI can accurately evaluate myocardial structure, ventricular function, and myocardial tissue characteristics in mouse models of CVD, providing powerful in vivo evidence for investigating cardiac remodeling and functional impairment (Zhao et al. 2026).

This study aimed to comprehensively determine the impact of calpain‐1 deficiency on both atherosclerotic plaque development and cardiac functional remodeling in ApoE‐deficient mice and to elucidate the underlying molecular mechanisms using integrated biochemical, 9.4 T small‐animal MRI, and Data‐Independent Acquisition (DIA)‐based proteomic approaches.

## 2 Materials and Methods

### 2.1 Chemicals and Reagents

Oil Red O (ORO), hematoxylin and eosin (H&E), and lipid profile kits were from Beijing Solarbio Company (Beijing, China). Kits for serum eNOS activity and NO content were purchased from Nanjing Jincheng Bioengineering Company (Nanjing, China). Antibodies for SR‐A, LXR‐α, and ABCA1 were purchased from Hangzhou UpingBio Company (Hangzhou, China). Antibodies for CD36, PPAR‐γ, eNOS, and GAPDH were the products of Beijing Solarbio Company (Beijing, China). The standard diet (SD) and HFD comprised 5% fat and 1.25% cholesterol, were sourced from the Weitonglihua Experimental Animal Center (Beijing, China).

### 2.2 Animals and Groups

Animal Ethics Committee of Lanzhou University Second Hospital approved the protocol (No. D2022‐191). C57 mice were provided by Animal Experiment Center at Lanzhou University (Lanzhou, China). ApoE KO and Calpain‐1 KO mice were obtained from Jackson Laboratories (Bar Harbor, Maine, USA). ApoE KO mice were crossbred with Calpain‐1 KO mice to obtain homozygous A × C DKO mice, which was identified genetically as described previously (Shi et al. 2025). The eight‐week‐old male mice were divided into three groups: C57 mice fed with SD; ApoE KO and A × C DKO mice were fed with HFD, respectively. Animals were randomly allocated to experimental groups to minimize selection bias, and all mice were maintained under identical environmental conditions throughout the study. All mice were fed SD or HFD for 8 weeks. All mice were maintained under conditions of a 12‐h light/dark cycle, a humidity of 55% ± 5% and temperature of 23°C ± 2°C, having free access to water and food.

### 2.3 Cardiac MRI

MRI was performed using a 9.4 T small‐animal MRI system (uMR 9.4 T, Shanghai United Imaging Healthcare Co. Ltd., China). Mice were anesthetized with isoflurane (1%–2%) and positioned supine on a dedicated animal imaging cradle with electrocardiographic and respiratory monitoring. Cine images were acquired using a gradient‐echo sequence with retrospective electrocardiogram (ECG) gating to obtain dynamic cardiac images throughout the cardiac cycle. Short‐axis slices covering the entire left ventricle and long‐axis views were obtained to evaluate cardiac morphology and function. Cardiac functional parameters were analyzed using the small‐animal cardiac analysis module of the uWS‐MR workstation. Endocardial borders of the left ventricle were manually delineated at end‐diastole and end‐systole across the short‐axis cine stack. Left ventricular end‐diastolic volume (LVEDV) and end‐systolic volume (LVESV) were calculated using the Simpson method. Left ventricle stroke volume (LVSV), ejection fraction (LVEF), and cardiac output (CO) were automatically derived by the software.

### 2.4 Sample Collection and Atherosclerotic Lesion Evaluation

Atherosclerotic plaque evaluation was conducted using the methodology previously described (Gu et al. 2023; Shi et al. 2025; Yin et al. 2017). Briefly, at the end of the experiment, mice were euthanized and blood was collected, from which serum was separated for biochemical analysis. After euthanasia, the thoracic cavity was opened and the heart, aortic arch, and brachiocephalic artery were exposed for morphological observation of lipid deposition inside the artery. The frozen cross sections of the aortic root were used for staining with ORO and H&E. The images were analyzed using ImageJ software, expressed in square micrometers (μm^2^). Both thoracic and abdominal aortas were isolated and immediately frozen in liquid nitrogen and then stored at −80°C for western blotting assay and DIA proteomics analysis.

### 2.5 Levels of Lipid Profile and Glucose in the Serum

The contents of Total cholesterol (TC), triglycerides (TG), low‐density lipoprotein cholesterol (LDL‐C), high‐density lipoprotein cholesterol (HDL‐C), and glucose in serum were measured using commercial assay kits purchased from Nanjing Jiancheng Bioengineering Institute (Nanjing, China) as described previously (Shi et al. 2025). Briefly, TC was determined by the cholesterol oxidase–peroxidase method, TG by the glycerol phosphate oxidase–peroxidase method, glucose by the glucose oxidase method, and LDL‐C and HDL‐C by enzymatic colorimetric assays following selective precipitation or direct assay procedures. Absorbance was measured with a microplate reader or an automated biochemical analyzer, and the concentrations were calculated based on the corresponding standard curves.

### 2.6 Contents of NO and Activity of eNOS in Serum

The NO level and eNOS activity in serum were determined using commercial assay kits purchased from Nanjing Jiancheng Bioengineering Institute (Nanjing, China) as described previously (Shi et al. 2025). Briefly, NO levels are quantified via the stable metabolite nitrite (NO_2_^−^) and nitrate (NO_3_^−^) using the Griess reaction and the reaction products were measured at 540 nm. eNOS enzymatic activity is measured via conversion of L‐arginine to L‐citrulline using a colorimetric assay and the reaction product was detected at 540 nm.

### 2.7 Protein Expression in the Aorta Evaluated by Western Blotting

For aortic tissue analysis, homogenize samples in RIPA buffer with protease/phosphatase inhibitors, quantify protein via BCA assay, and load 20–30 μg per lane onto SDS‐PAGE gels (10%–12%). After electrophoresis, transfer proteins to PVDF membranes using wet transfer (100 V, 60–90 min), block with 5% non‐fat milk/TBST (1 h, RT), then incubate overnight at 4°C with primary antibodies diluted in 5% BSA/TBST: CD68 (1:1000), SR‐A (1:1000), CD36 (1:1000), PPAR‐γ (1:2000), LXR‐α (1:2000), and GAPDH (1:5000) as the internal reference. Wash membranes, incubate with HRP‐conjugated secondary antibody (1:5000, 1 h, RT), develop using ECL substrate, and quantify band densities normalized to GAPDH via chemiluminescence imaging.

### 2.8 Proteomic Profiling and Bioinformatics of Aortic Tissue

Aortic tissue samples are snap‐frozen in liquid N_2_, homogenized in 8 M urea lysis buffer, reduced/alkylated (10 mM DTT, 55 mM iodoacetamide), and digested with trypsin/Lys‐C (1:25 w/w, 37°C, overnight). Peptides were desalted (C18 StageTips) and analyzed via nano‐LC–MS/MS using a Q‐Exactive HF‐X mass spectrometer in data‐independent acquisition (DIA) mode: peptides separated on a 75‐μm C18 column with a 120‐min gradient (5%–35% acetonitrile/0.1% formic acid); full‐scan MS1 (350–1500 m/z, *R* = 60,000) followed by variable‐window MS2 (*R* = 30,000, NCE = 28%). Raw DIA files are processed using Spectronaut (v18) or DIA‐NN (v1.8) against a species‐specific spectral library (generated from pooled samples via DDA), enabling label‐free quantification (LFQ) with FDR ≤ 1%. Bioinformatics analysis includes differential expression (limma, |log_2_FC| > 0.5, adj. *p* < 0.05), functional enrichment (clusterProfiler for GO/KEGG pathways), with key atherosclerosis‐related pathways (e.g., extracellular matrix (ECM) remodeling, inflammation).

### 2.9 Statistical Analysis

The data are presented as mean ± standard deviation (SD). One‐way analysis of variance (ANOVA) followed by Tukey's post hoc test was used for multiple group comparisons using the statistical software packages SPSS 27.0 and GraphPad Prism 9.5. *p* < 0.05 was considered to indicate a statistically significant difference.

## 3 Results

### 3.1 Calpain‐1 KO Attenuates Atherosclerotic Plaques and Downregulates Protein Expression of CD68 in the Aorta Without Affecting the Body Weight

Body weight increased progressively in all groups during the 8‐week period, with ApoE KO and A × C DKO mice showing significantly higher body weight than C57 mice, while no significant difference was observed between ApoE KO and A × C DKO mice (Figure 1A). Morphological examination of the aortic arch and brachiocephalic artery revealed complete absence of atherosclerotic plaques in C57 wild‐type mice. In contrast, ApoE KO mice exhibited significant plaque formation in these vascular regions (Figure 1B). This plaque presence was markedly decreased in A × C DKO mice compared to the ApoE KO group. Similarly, quantitative analysis of cross‐section plaque area showed no detectable plaques in C57 controls, a significant presence in ApoE KO mice, and a distinct decrease in the A × C DKO group relative to ApoE KO (Figure 1C,D). Immunologically, assessment of CD68 protein expression (a marker for plaque foam cells) demonstrated that the expression significantly increased in ApoE KO mice versus C57 controls, but significantly decreased in A × C DKO mice versus ApoE KO mice (Figure 1E,F). Collectively, these findings across morphological, quantitative, and immunological parameters consistently show that ApoE deficiency drives atherosclerotic plaque development and inflammation, while concurrent calpain‐1 knockout (A × C DKO) mitigates these effects.

**FIGURE 1:**
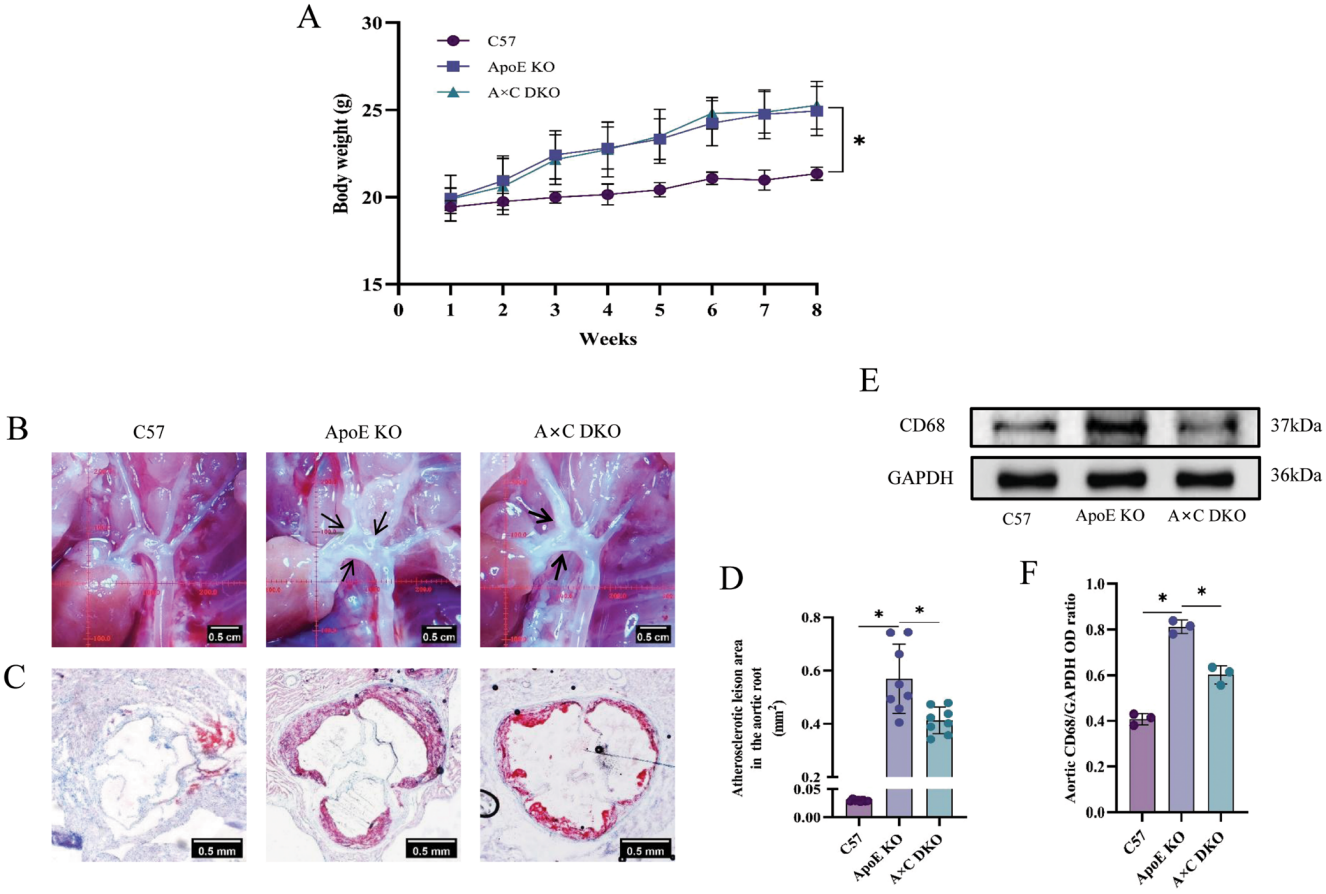
Calpain‐1 KO attenuates atherosclerotic plaques and downregulates protein expression of CD68 in the aorta without affecting the body weight. Eight‐week‐old mice were divided into three groups: C57 mice fed SD (C57 group); ApoE KO mice fed HFD (ApoE KO group); A × C DKO fed HFD (A × C DKO group). All mice were fed a SD or HFD for 8 weeks. (A) Body weight. (B) Morphological examination of the aortic arch and brachiocephalic artery with arrows indicating the atherosclerotic lesion. Scale bars = 0.5 mm. (C) Frozen aortic root sections stained with ORO. Scale bars = 0.5 mm. (D) The statistical results from panel B. (E, F) Aortic CD68 protein expression. Data are presented as mean ± SD, *n* = 8 for A‐D, *n* = 3 for E, F. *p < 0.05 was considered statistically significant.

### 3.2 Calpain‐1 KO Attenuates Cardiac Systolic Dysfunction

Cardiac structure and function were assessed using cine MRI. Representative short‐axis and long‐axis images at end systole and end diastole are shown in Figure 2A,B. Compared with C57 control mice, ApoE KO mice exhibited evident ventricular dilation, characterized by increased LVESV (Figure 2C) and LVEDV (Figure 2D). Consistently, LVEF (Figure 2E) was markedly reduced, indicating impaired systolic function. In contrast, A × C DKO mice showed partial normalization of ventricular volumes, with decreased LVESV and moderately reduced LVEDV compared with ApoE KO mice. Correspondingly, LVEF (Figure 2E) was significantly improved in A × C DKO mice, suggesting enhanced contractile performance. LVSV (Figure 2F) and CO (Figure 2G) were significantly increased in A × C DKO mice. These findings indicate that calpain‐1 deficiency alleviates ventricular remodeling and improves cardiac systolic function in ApoE‐deficient mice.

**FIGURE 2:**
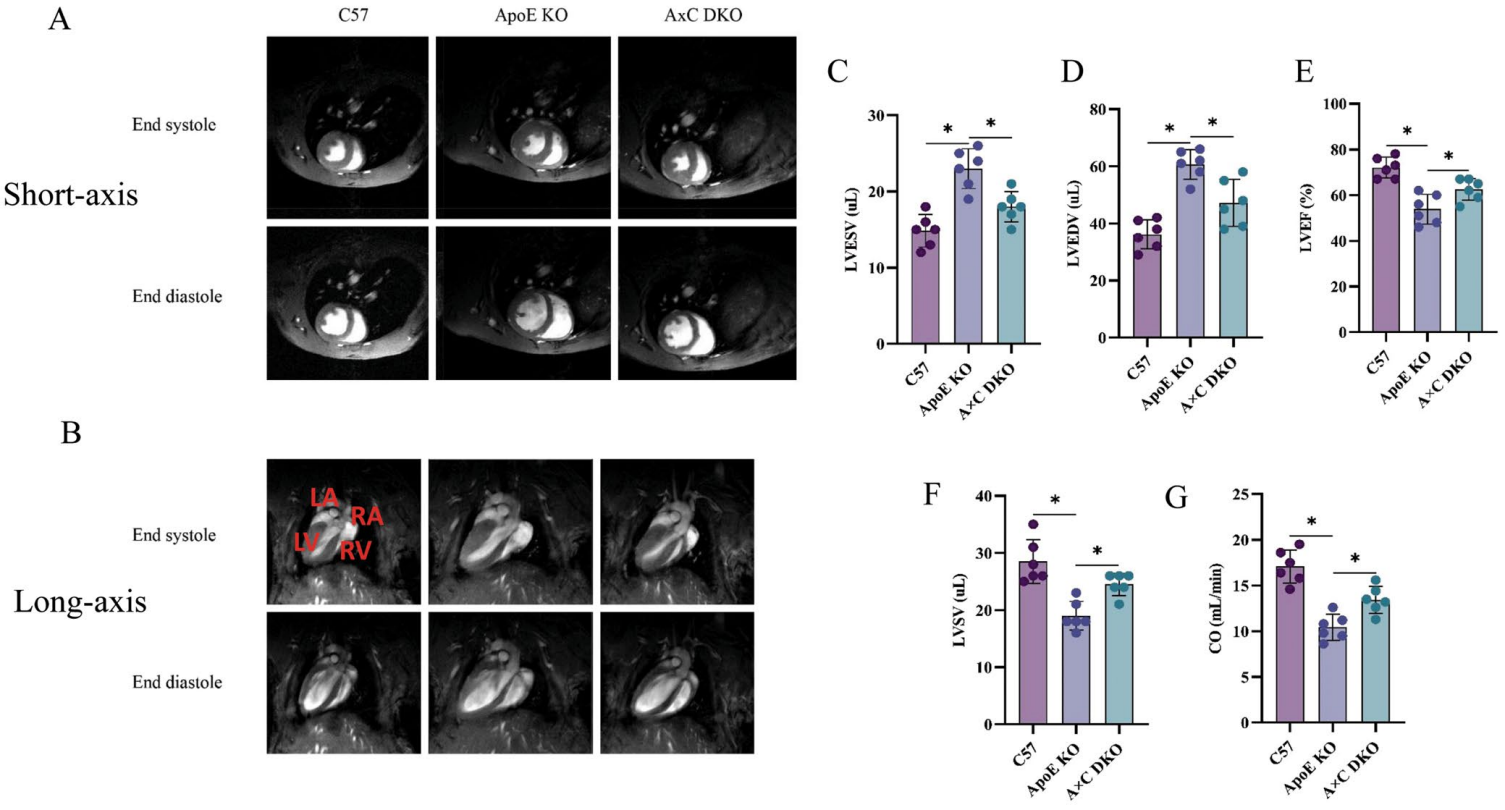
Calpain‐1 KO attenuates cardiac systolic dysfunction. Eight‐week‐old mice were divided into three groups: C57 mice fed SD (C57 group); ApoE KO mice fed HFD (ApoE KO group); A × C DKO fed HFD (A × C DKO group). All mice were fed a SD or HFD for 8 weeks. Cardiac structure and function were assessed using cine MRI. LA, left atrium; LV, left ventricle; RA, right atrium; RV, right ventricle. (A, B) Representative short‐axis and long‐axis images at end systole and end diastole. (C) LVESV. (D) LVEDV. (E) LVEF. (F) LVSV. (G) CO. Data are presented as mean ± SD, *n* = 6 for A–G. *p < 0.05 was considered statistically significant.

### 3.3 Calpain‐1 KO Regulates Aortic Protein Expression Associated With Atherosclerotic Foamy Plaques

Analysis of scavenger receptor and cholesterol regulatory pathways in aorta showed that protein expression of SR‐A (Figure 3A,B) and CD36 (Figure 3A,C) significantly increased in ApoE KO mice versus C57 wild‐type controls, indicating enhanced foam cell uptake in plaques. However, A × C DKO mice showed a marked decrease in SR‐A and CD36 expression compared to ApoE KO. For cholesterol efflux regulators, the protein expression of PPAR‐γ (Figure 3A,D) and LXR‐α (Figure 3A,E) decreased significantly in ApoE KO mice compared to C57 controls, reflecting impaired reverse cholesterol transport. In contrast, A × C DKO mice exhibited a significant increase in PPAR‐γ and LXR‐α expression versus ApoE KO mice. Taken together, these results indicate that calpain‐1 ablation in ApoE‐deficient mice attenuates foam cell receptor expression while restoring cholesterol efflux machinery.

**FIGURE 3:**
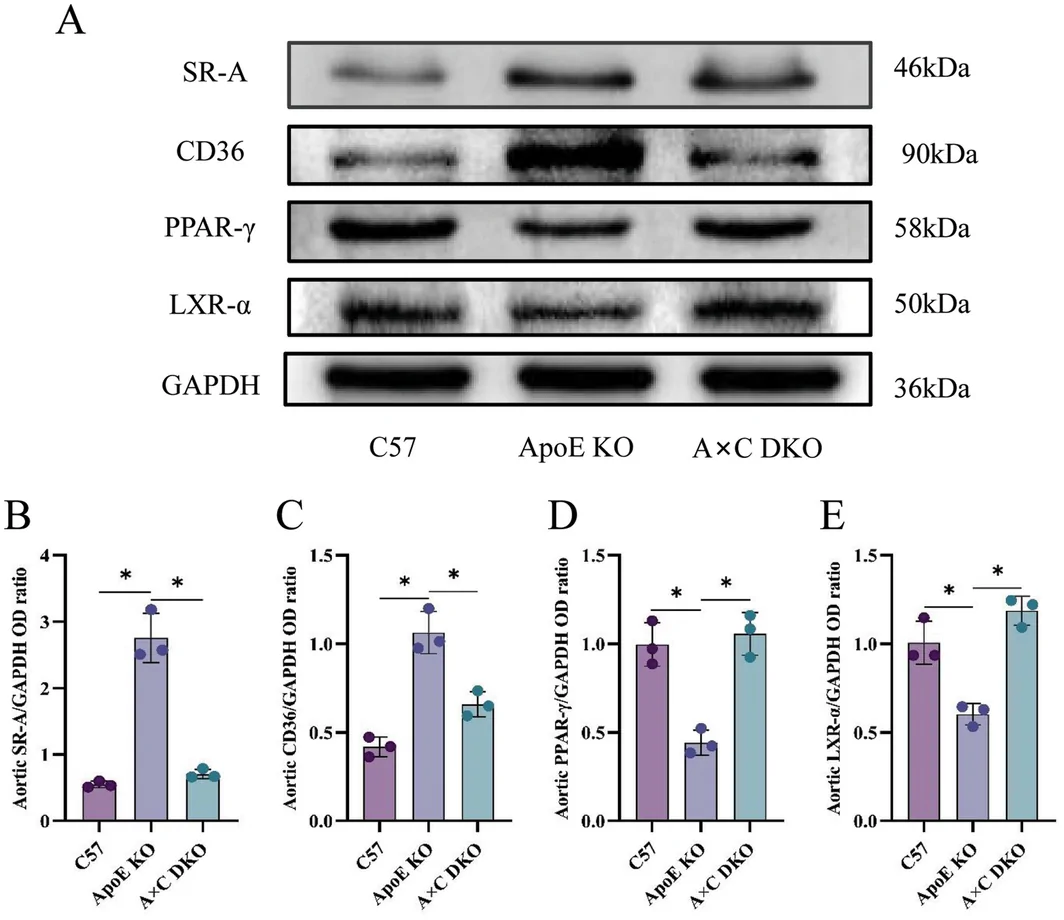
Calpain‐1 KO regulates aortic protein expression associated with atherosclerotic foamy plaques. Eight‐week‐old mice were divided into three groups: C57 mice fed SD (C57 group); ApoE KO mice fed HFD (ApoE KO group); A × C DKO fed HFD (A × C DKO group). All mice were fed a SD or HFD for 8 weeks. (A, B) SR‐A protein expression. (A, C) CD36 protein expression. (A, D) PPAR‐γ protein expression. (A, E) LXR‐α protein expression. Data are presented as mean ± SD, *n* = 3. *p < 0.05 was considered statistically significant.

### 3.4 Calpain‐1 KO Had No Significant Effect on Lipid Profile and Glucose Level in Serum

Measurement of serum lipid profiles and glucose level revealed that serum TC (Figure 4A), TG (Figure 4B), LDL‐C (Figure 4C) show significant increases in ApoE KO mice compared to C57 wild‐type controls accompanied by reduced HDL‐C (Figure 4D), confirming expected dyslipidemia in the ApoE knockout model. In addition, glucose level was increased in ApoE KO mice. However, these parameters in A × C DKO mice remained unchanged relative to ApoE KO mice. These findings indicate that calpain‐1 deletion does not significantly alter systemic lipid or glucose levels in ApoE‐deficient mice. These findings suggest that calpain‐1 KO attenuates atherosclerosis independent of serum lipid profiles and glucose level.

**FIGURE 4:**
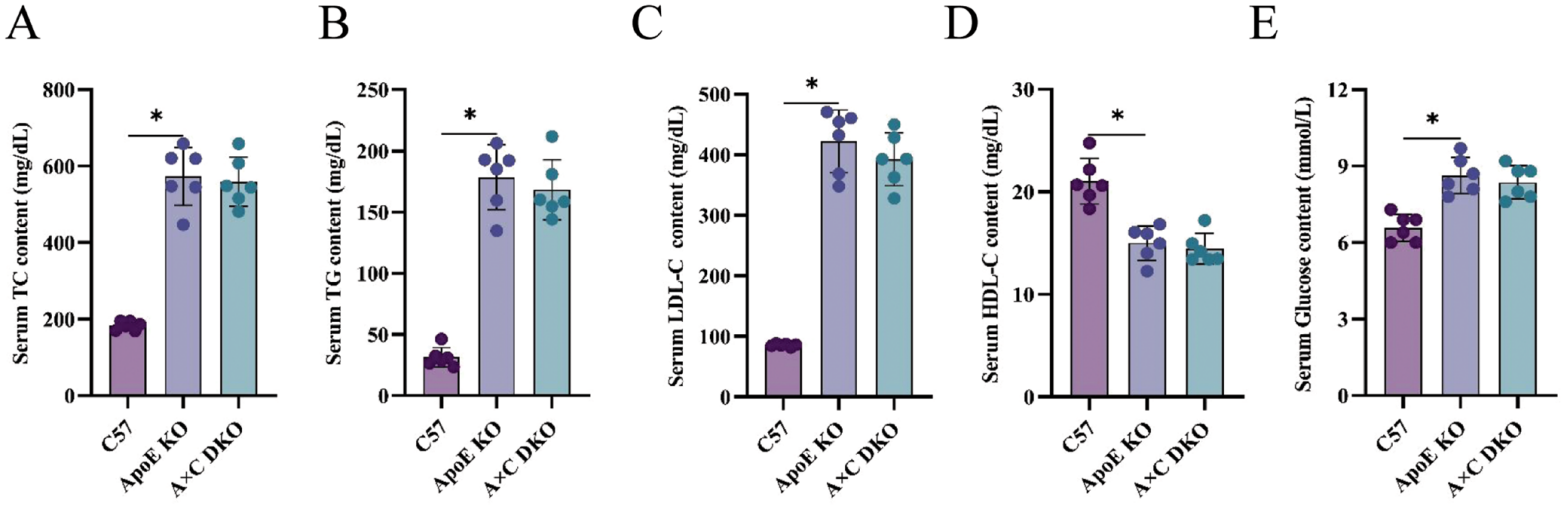
Calpain‐1 KO had no significant effect on lipid profile and glucose level in serum. Eight‐week‐old mice were divided into three groups: C57 mice fed SD (C57 group); ApoE KO mice fed HFD (ApoE KO group); A × C DKO fed HFD (A × C DKO group). All mice were fed a SD or HFD for 8 weeks. (A) TC content. (B) TG content. (C) LDL‐C content. (D) HDL‐C content. (E) Glucose content. Data are presented as mean ± SD, *n* = 8 for A–F, *p < 0.05 was considered statistically significant.

### 3.5 Calpain‐1 KO Increases NO Content, eNOS Activity in Serum, and Protein Expression of Aortic eNOS

Detection of endothelial function markers revealed that compared to C57 wild‐type controls, ApoE KO mice exhibited significant decreases in serum NO content (Figure 5A), eNOS activity (Figure 5B), and aortic eNOS protein expression (Figure 5C,D), indicating severe endothelial dysfunction. However, A × C DKO mice demonstrated significant increases in all three parameters, serum NO level, eNOS activity, and aortic eNOS protein expression, relative to ApoE KO mice. These findings indicated that attenuation of atherosclerosis by calpain‐1 KO might be attributed to the improvement of endothelial function.

**FIGURE 5:**
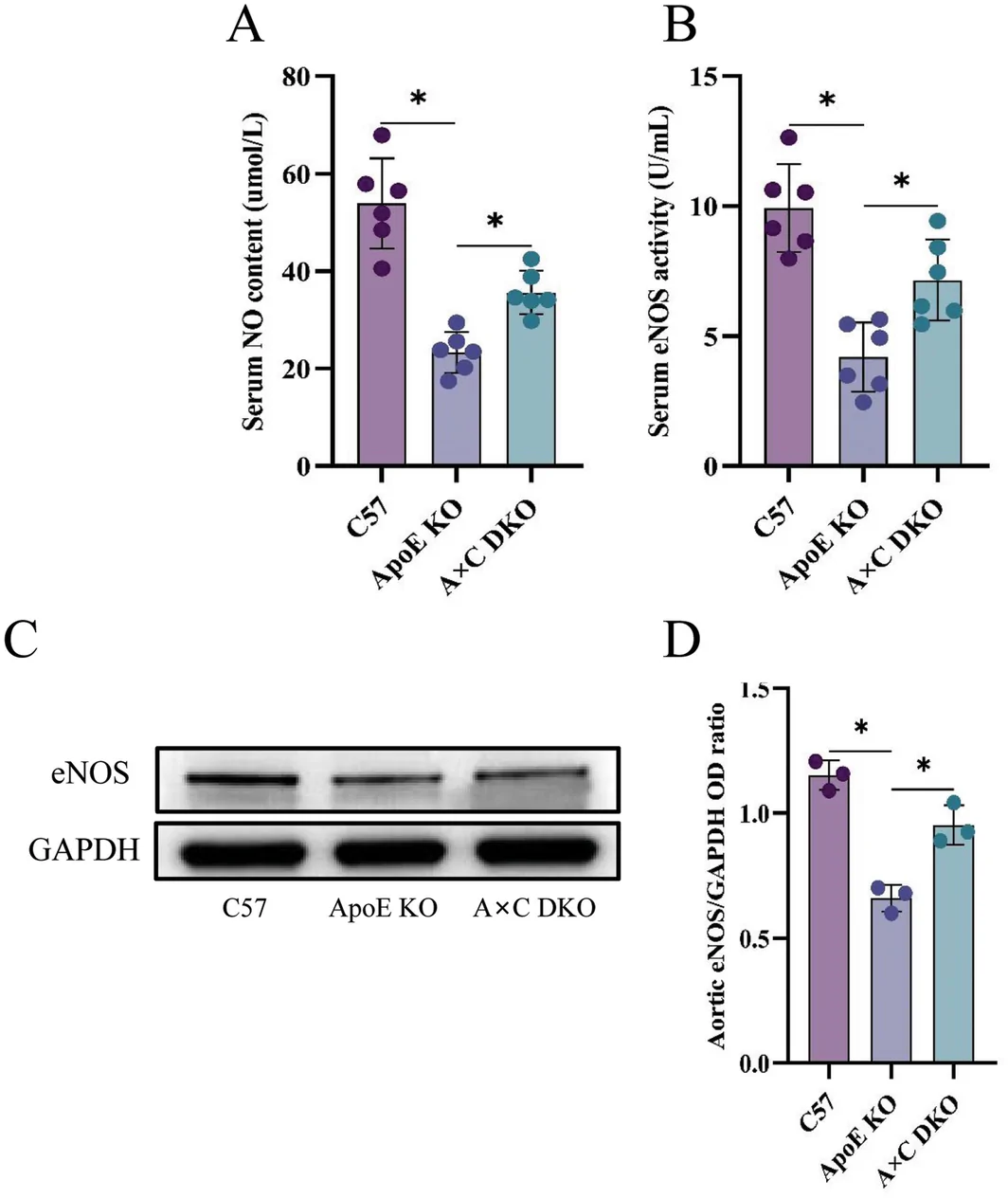
Calpain‐1 KO increases NO content, eNOS activity in serum and protein expression of aortic eNOS. Eight‐week‐old mice were divided into three groups: C57 mice fed SD (C57 group); ApoE KO mice fed HFD (ApoE KO group); A × C DKO fed HFD (A × C DKO group). All mice were fed a SD or HFD for 8 weeks. (A) NO content in serum. (B) eNOS activity. (C, D) eNOS protein expression. Data are presented as mean ± SD, *n* = 8 for A, B, *n* = 3 for C, D. *p < 0.05 was considered statistically significant.

### 3.6 Effects of Calpain‐1 KO on Aortic Proteomics

#### 3.6.1 Differentially Expressed Proteins Determined by DIA

DIA proteomic analysis identified 5721 proteins with quantitative information in three groups. Compared with the C57 group, the ApoE KO group revealed a total of 4252 differentially expressed proteins, having 1953 up‐regulated and 2299 down‐regulated (Figure 6A). Compared with the ApoE KO group, the A × C DKO demonstrated a total of 1119 differentially expressed proteins, having 535 up‐regulated and 579 down‐regulated (Figure 6B). Among them, 307 proteins were found simultaneously up‐regulated in the ApoE KO versus C57 groups and down‐regulated in the A × C DKO versus ApoE KO groups (Figure 6C). Similarly, 141 proteins were found simultaneously down‐regulated in the ApoE KO versus C57 groups and up‐regulated in the A × C DKO versus ApoE KO groups (Figure 6D). The function analysis demonstrated that these proteins participate in the process of enzymatic triggers of foam cell formation, cytoskeleton‐mediated macrophage recruitment, endothelial barrier function and vascular homeostasis, ECM destabilization and protease dysregulation, and redox imbalance and mitochondrial dysfunction.

**FIGURE 6:**
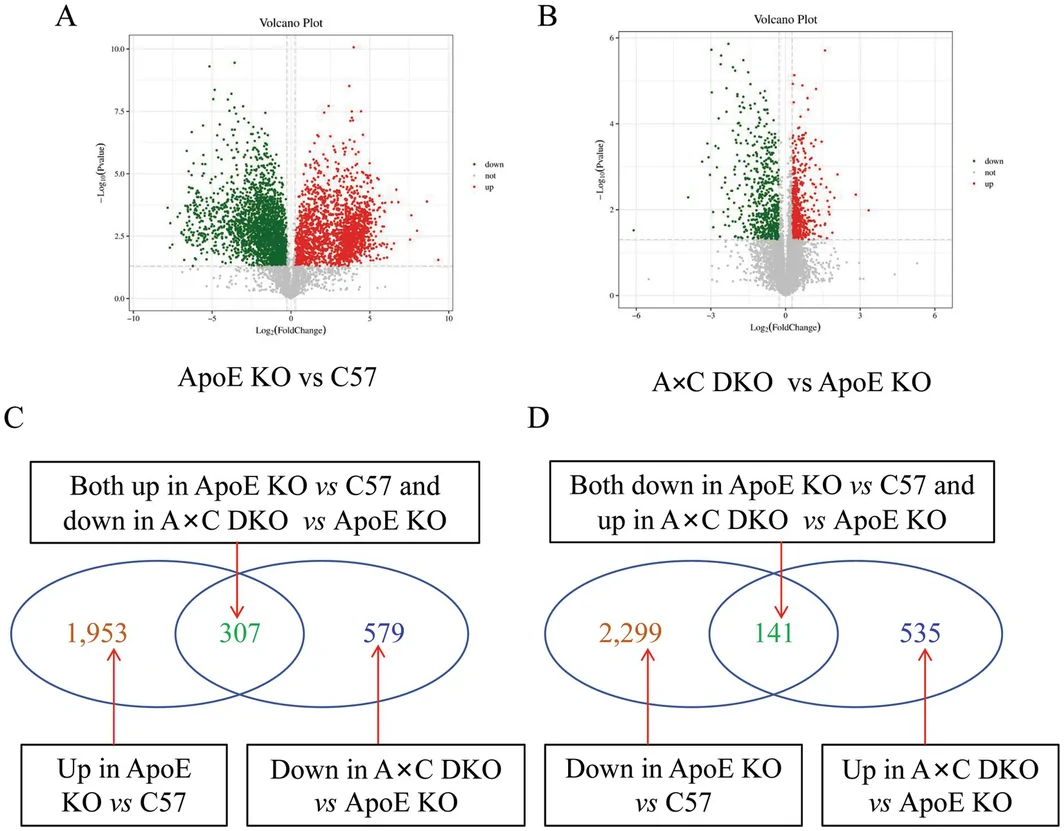
Differentially expressed proteins determined by using DIA proteomic analysis. Eight‐week‐old mice were divided into three groups: C57 mice fed SD (C57 group); ApoE KO mice fed HFD (ApoE KO group); A × C DKO fed HFD (A × C DKO group). All mice were fed a SD or HFD for 8 weeks. (A) Volcano plot comparing ApoE KO group with C57 group. (B) Volcano plot comparing A × C DKO group with ApoE KO group. (C, D) Venn diagram of overlapping DEPs. Data are expressed as the means ± SD. *n* = 3. *p < 0.05 was considered statistically significant.

#### 3.6.2 Calpain‐1 KO Attenuates Enzymatic Triggers of Foam Cell Formation

DIA proteomics analysis of lipid‐processing enzymes showed that, compared to C57 controls, ApoE KO mice exhibited increased expression of the triglyceride hydrolase LPL (Figure 7A), which enhances fatty acid release for foam cell formation, and elevated cholesterol acyltransferase SOAT1 (Figure 7B), promoting cholesterol esterification and lipid droplet accumulation in macrophages. On the contrary, A × C DKO mice showed decreased expression of both LPL and SOAT1 compared to ApoE KO mice, reducing triglyceride‐derived fatty acid availability and suppressing cholesterol ester storage. The findings demonstrate calpain‐1 ablation downregulates critical enzymatic drivers of intracellular lipid accumulation, thereby attenuating foam cell development in ApoE‐deficient atherosclerosis.

**FIGURE 7:**
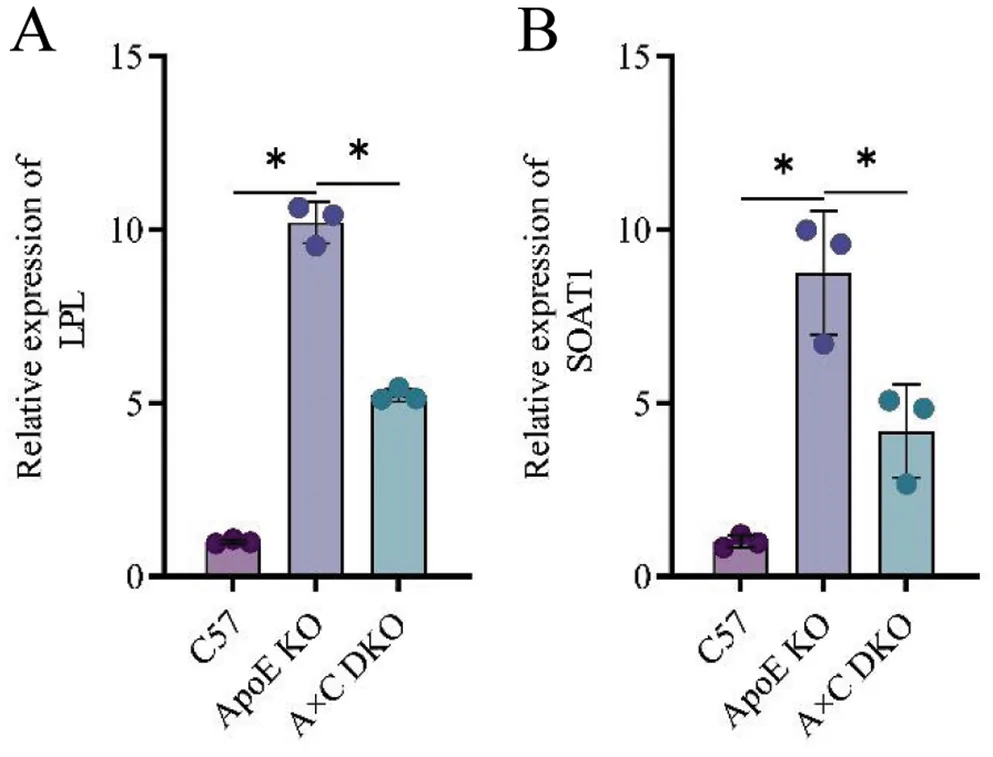
Calpain‐1 KO attenuates enzymatic triggers of foam cell formation. Eight‐week‐old mice were divided into three groups: C57 mice fed SD (C57 group); ApoE KO mice fed HFD (ApoE KO group); A × C DKO fed HFD (A × C DKO group). All mice were fed a SD or HFD for 8 weeks. (A) LPL protein expression. (B) SOAT1 protein expression. Data are expressed as the means ± SD. *n* = 3. *p < 0.05 was considered statistically significant.

#### 3.6.3 Calpain‐1 KO Attenuates Cytoskeleton‐Mediated Macrophage Recruitment and Endothelial Permeability

DIA proteomics analysis of cytoskeletal dynamics revealed that, relative to C57 controls, ApoE KO mice exhibited increased expression of actin regulators (CFL1, LCP1) (Figure 8A,B), enhancing macrophage migration into developing plaques, and elevated myosin isoforms (MYO1E/1F) (Figure 8C,D), promoting endothelial barrier dysfunction and lipoprotein permeability. However, A × C DKO mice showed decreased expression of both actin regulators and myosins compared to ApoE KO mice. Taken together, these results indicate that calpain‐1 co‐deletion attenuates cytoskeleton‐mediated pro‐atherogenic processes, specifically reducing macrophage infiltration by suppressing actin regulators and improving endothelial integrity through myosin normalization in ApoE‐deficient atherosclerosis.

**FIGURE 8:**
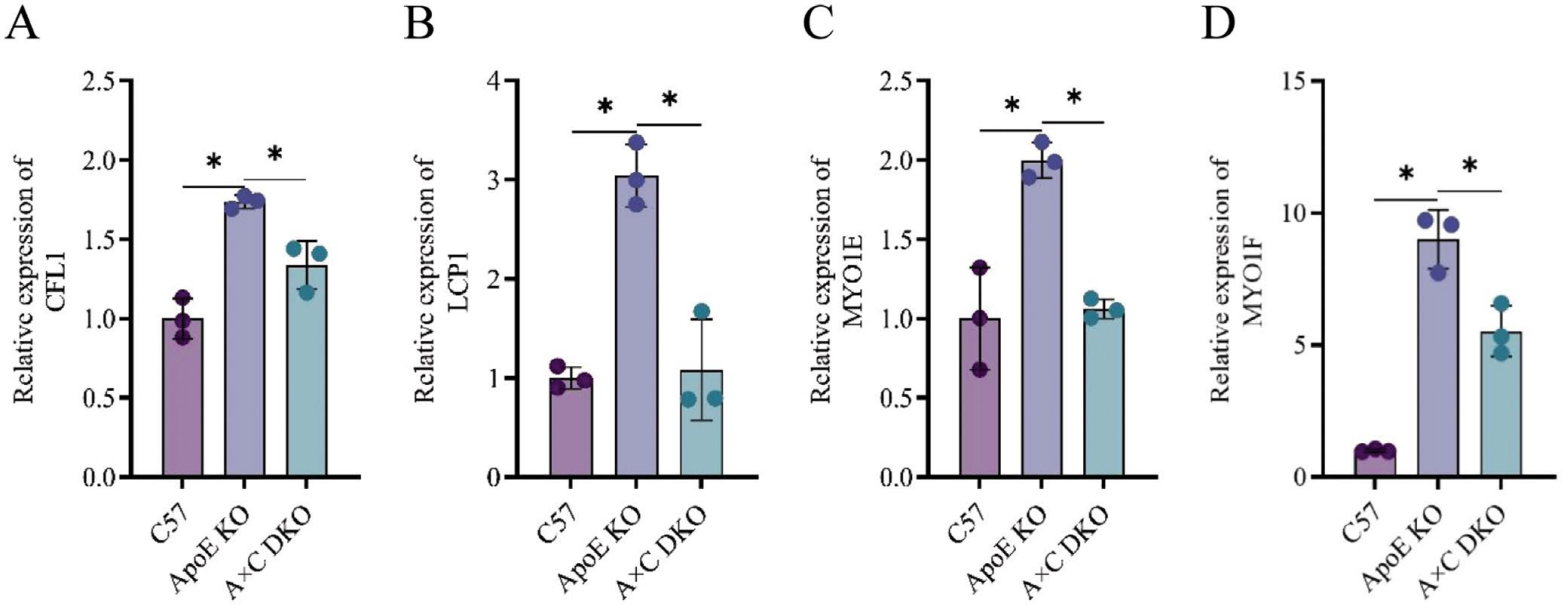
Calpain‐1 KO attenuates cytoskeleton‐mediated macrophage recruitment and endothelial permeability. Eight‐week‐old mice were divided into three groups: C57 mice fed SD (C57 group); ApoE KO mice fed HFD (ApoE KO group); A × C DKO fed HFD (A × C DKO group). All mice were fed a SD or HFD for 8 weeks. (A) CFL1 protein expression. (B) LCP1 protein expression. (C) MYO1E protein expression. (D) MYO1F protein expression. Data are expressed as the means ± SD. *n* = 3. *p < 0.05 was considered statistically significant.

#### 3.6.4 Calpain‐1 KO Restores Endothelial Barrier Function and Vascular Homeostasis

Analysis of endothelial and vascular integrity markers in DIA proteomics demonstrated that, compared to C57 controls, ApoE KO mice exhibited decreased expression of ITGA1 (Figure 9A) and CD9 (Figure 9B), resulting in compromised ECM adhesion (promoting lipid retention) and disrupted angiogenesis signaling (affecting neovascularization). On the contrary, A × C DKO mice showed increased expression of two markers in comparison with ApoE KO mice, indicating restoration of endothelial adhesion capabilities and normalized angiogenesis signaling. These results demonstrate that calpain‐1 KO rescues multiple facets of endothelial dysfunction in ApoE‐deficient atherosclerosis by restoring vascular integrity pathways.

**FIGURE 9:**
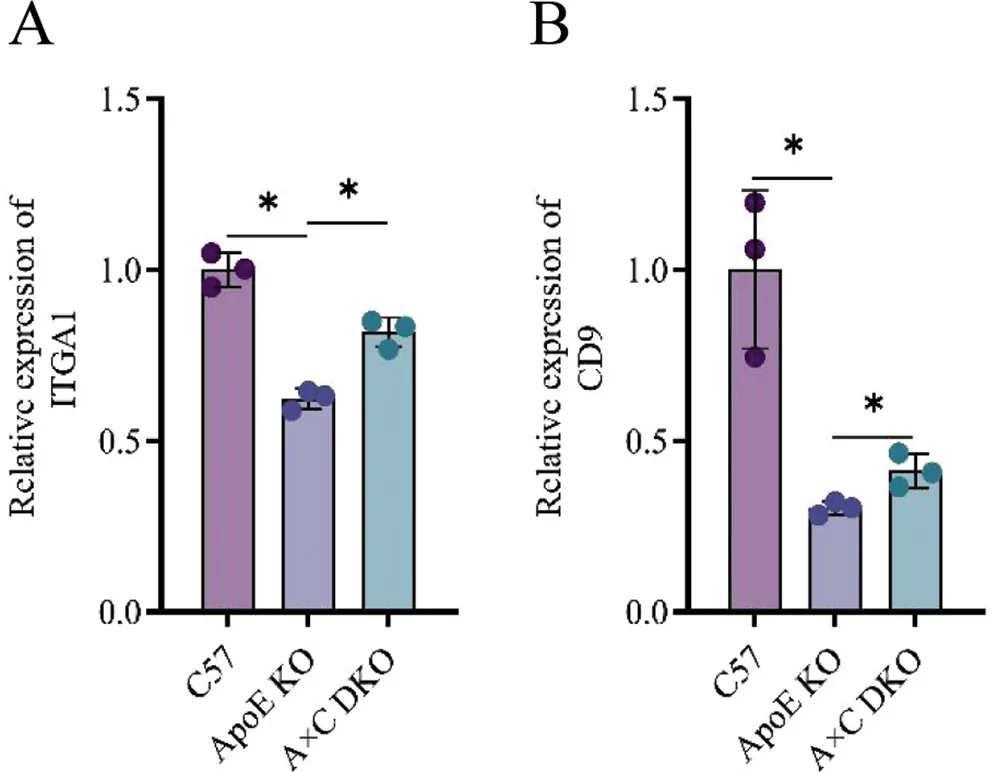
Calpain‐1 KO restores endothelial barrier function and vascular homeostasis. Eight‐week‐old mice were divided into three groups: C57 mice fed SD (C57 group); ApoE KO mice fed HFD (ApoE KO group); A × C DKO fed HFD (A × C DKO group). All mice were fed a SD or HFD for 8 weeks. (A) ITGA1 protein expression. (B) CD9 protein expression. Data are expressed as the means ± SD. *n* = 3. *p < 0.05 was considered statistically significant.

#### 3.6.5 Calpain‐1 KO Reverses ECM Destabilization and Protease Dysregulation in Atherosclerotic Plaques

Coordinated analysis of ECM remodeling and protease‐inhibitor systems in DIA proteomics revealed that, relative to C57 controls, ApoE KO mice exhibited increased collagen deposition (COL6A5, COL12A1) (Figure 10A,B) in fibrous caps, elevated matrix proteins (FN1, VTN) (Figure 10C,D) promoting lipid retention, upregulated adhesion molecules (VCAM1, CD44, ITGB2) (Figure 10E–G) enhancing leukocyte recruitment, augmented protease expression (MMP2/3/12/19) (Figure 10H–K) driving ECM degradation and plaque instability, and reduced serpin inhibitors (TIMP1) (Figure 10L) accelerating these destructive processes. However, A × C DKO mice showed significant reversal across all parameters: collagen/matrix/adhesion molecule expression decreased, protease activity declined, and serpin inhibitors (including TIMP1) increased relative to ApoE KO mice. This integrated increase‐then‐decrease trajectory in ECM/adhesion/protease markers with an inverse decrease‐then‐increase response in protective inhibitors demonstrates that calpain‐1 co‐deletion restores ECM homeostasis, attenuates proteolytic damage, and reduces plaque vulnerability in ApoE‐deficient atherosclerosis.

**FIGURE 10:**
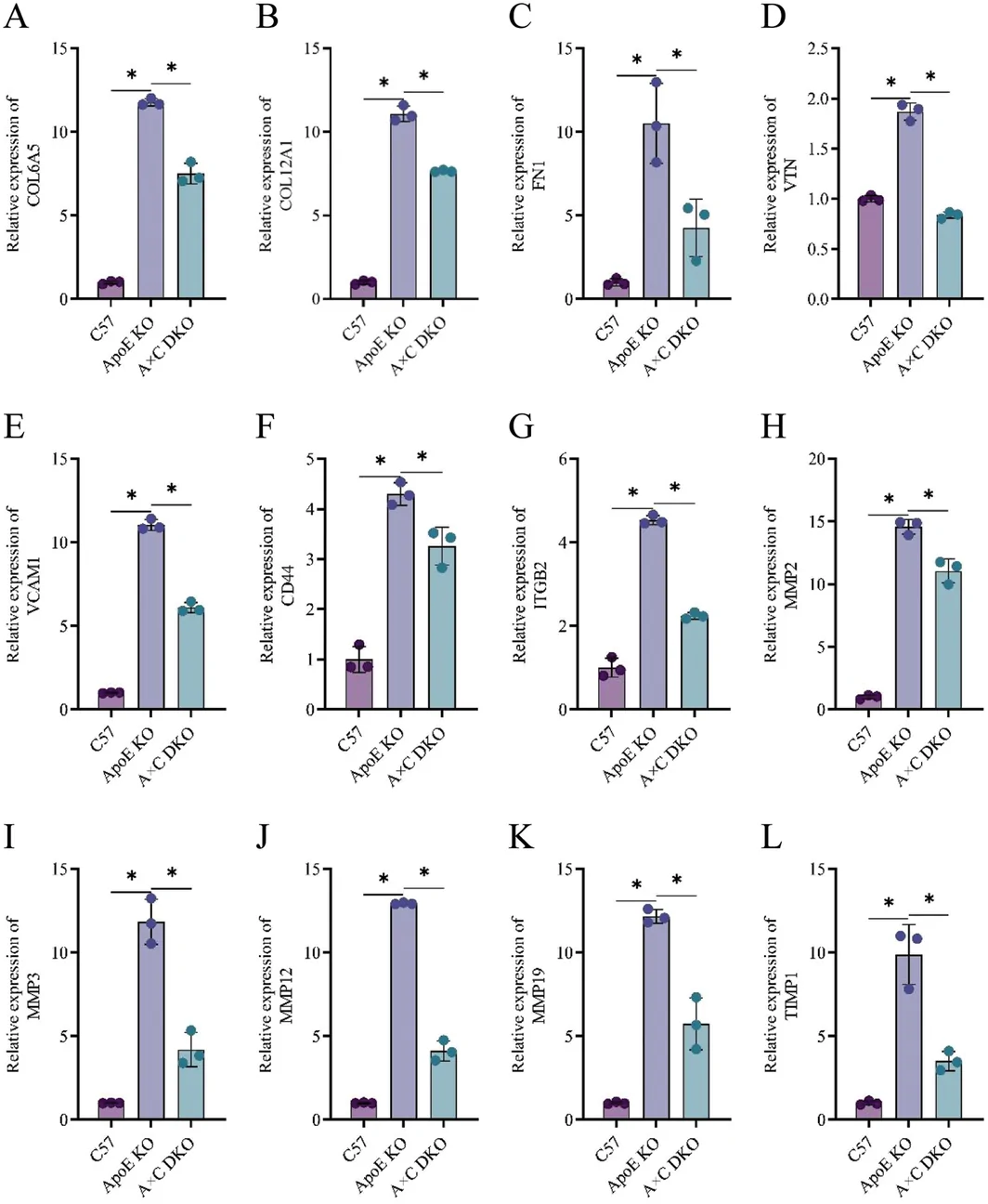
Calpain‐1 KO reverses ECM destabilization and protease dysregulation in atherosclerotic plaques. Eight‐week‐old mice were divided into three groups: C57 mice fed SD (C57 group); ApoE KO mice fed HFD (ApoE KO group); A × C DKO fed HFD (A × C DKO group). All mice were fed a SD or HFD for 8 weeks. (A) COL6A5 protein expression. (B) COL12A1 protein expression. (C) FN1 protein expression. (D) VTN protein expression. (E) VCAM1 protein expression. (F) CD44 protein expression. (G) ITGB2 protein expression. (H) MMP2 protein expression. (I) MMP3 protein expression. (J) MMP12 protein expression. (K) MMP19 protein expression. (L) TIMP1 protein expression. Data are expressed as the means ± SD. *n* = 3. *p < 0.05 was considered statistically significant.

#### 3.6.6 Calpain‐1 KO Coordinates Reversal of Redox Imbalance and Mitochondrial Dysfunction

DIA proteomics analysis of oxidative and mitochondrial pathways revealed distinct regulatory effects modulated by calpain‐1 co‐deletion. For pro‐oxidative enzymes (HPGDS, PAOX) (Figure 11A,B), ApoE KO mice showed increased expression compared to C57 controls, driving pro‐inflammatory eicosanoid production and ROS generation, while A × C DKO mice exhibited decreased expression in comparison to ApoE KO mice. On the contrary, antioxidant defense marker (GSTM6) (Figure 11C) and mitochondrial function regulators (PDHA1, CS) (Figure 11D,E) demonstrated decreased expression in ApoE KO mice relative to C57 controls, resulting in elevated mitochondrial ROS, reduced oxidized LDL detoxification, impaired pyruvate oxidation, diminished TCA cycle flux, and compromised electron transport chain efficiency. Critically, A × C DKO mice displayed increased expression of all antioxidant and mitochondrial markers versus ApoE KO mice, restoring redox balance and mitochondrial bioenergetics. These results demonstrate calpain‐1 ablation simultaneously suppresses oxidative catalysts while rescuing cellular defense systems in ApoE‐deficient atherosclerosis.

**FIGURE 11:**
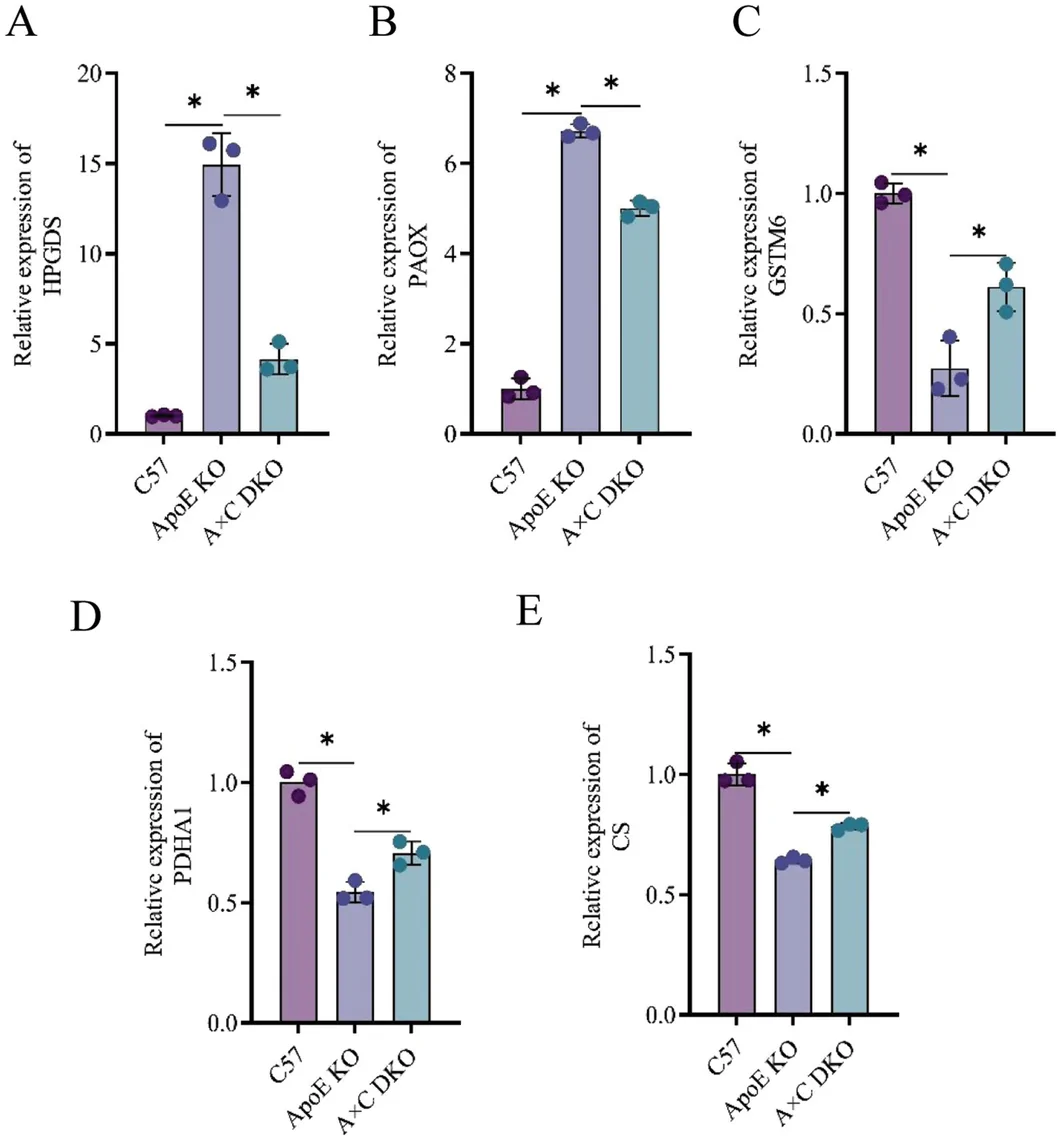
Calpain‐1 KO coordinates reversal of redox imbalance and mitochondrial dysfunction. Eight‐week‐old mice were divided into three groups: C57 mice fed SD (C57 group); ApoE KO mice fed HFD (ApoE KO group); A × C DKO fed HFD (A × C DKO group). All mice were fed a SD or HFD for 8 weeks. (A) HPGDS protein expression. (B) PAOX protein expression. (C) GSTM6 protein expression. (D) PDHA1 protein expression. (E) CS protein expression. Data are expressed as the means ± SD. *n* = 3. *p < 0.05 was considered statistically significant.

## 4 Discussion

The present study provides evidence that calpain‐1 contributes to the progression of atherosclerosis and cardiovascular dysfunction in ApoE‐deficient mice. Using a combination of histological evaluation, biochemical analysis, cardiac MRI, and DIA‐based proteomics, we demonstrate that genetic deletion of calpain‐1 significantly attenuates atherosclerotic plaque formation and improves cardiac systolic function. Importantly, these protective effects occurred without significant alterations in systemic lipid profiles or glucose levels, indicating that calpain‐1 regulates atherosclerosis through lipid‐independent mechanisms involving endothelial protection, suppression of foam cell formation, restoration of extracellular matrix homeostasis, and improvement of mitochondrial metabolism.

The imperative to identify novel targets like calpain‐1 stems from atherosclerosis remaining the primary underlying pathology of CVD, the leading global cause of mortality (Zhang, Cheng, and Jian 2025). Despite advances in lipid‐lowering therapies, residual inflammatory and metabolic risks drive persistent plaque progression and clinical events in > 30% of treated patients (Rai 2024). Our data pinpoint several key mechanisms underlying this protection, which were analyzed and discussed in the following.

Foam cell formation is a key initiating event in atherosclerosis and is largely driven by the imbalance between lipid uptake and cholesterol efflux in macrophages (Huang et al. 2026). In the present study, calpain‐1 KO significantly downregulated the scavenger receptors SR‐A and CD36, pivotal for modified LDL uptake into macrophages (Makinen et al. 2010; Perun et al. 2022), while concurrently upregulating the cholesterol efflux machinery (PPAR‐γ, LXR‐α) (Marasinghe et al. 2024). Proteomics further revealed normalization of key lipid‐processing enzymes: reduced LPL (limiting fatty acid release from triglycerides) and decreased SOAT1 (reducing cholesterol esterification and droplet accumulation) (Melton et al. 2019; Wu et al. 2018). This coordinated suppression of uptake pathways and enhancement of efflux pathways effectively attenuates the core process of foam cell generation, a cornerstone of early atherogenesis. Consistently, one study has revealed that myeloid ACAT1/SOAT1 KO attenuates pro‐inflammatory responses in macrophages and protects against atherosclerosis in a model of advanced lesions (Melton et al. 2019). Another study reported that SOAT1 deficiency attenuates atherosclerosis by regulating inflammation and cholesterol transportation via HO‐1 pathway (Wu et al. 2018).

Endothelial dysfunction is widely recognized as a critical early event in atherogenesis (Prasannan et al. 2026). Reduced NO bioavailability leads to impaired vasodilation, increased vascular inflammation, and enhanced leukocyte adhesion (Long et al. 2026). In the present study, ApoE deficiency severely impaired endothelial function, characterized by decreased eNOS activity, reduced serum NO bioavailability, and diminished aortic eNOS expression, hallmarks of endothelial dysfunction promoting inflammation and vasoconstriction (Hayashi et al. 2004; Yu et al. 2018). Calpain‐1 ablation robustly reversed all these parameters. Proteomics corroborated this functional recovery, showing increased expression of markers critical for endothelial integrity (ITGA1 for ECM adhesion, PRDX3 for ROS scavenging/NO protection, CD9 for angiogenesis signaling) and decreased expression of actin regulators (promoting macrophage migration) and myosin isoforms (causing endothelial barrier dysfunction) (Kim and Choi 2020; Su et al. 2024). This demonstrates calpain‐1 KO rescues endothelial NO production and barrier function, crucial for vascular homeostasis. In comparison, the previous results supported that CD9 expression may play an important role in the vascular senescence and pathogenesis of atherosclerosis (Kim and Choi 2020).

Atherosclerotic plaque progression is closely associated with ECM remodeling and proteolytic degradation (Zhang, Feng, and Kong 2024). Excessive matrix metalloproteinase (MMP) activity leads to fibrous cap thinning and increased plaque vulnerability. Our proteomic analysis unveiled a profound reversal of ECM destabilization and proteolytic imbalance in DKO mice. ApoE deficiency increased collagen types, matrix proteins (FN1, VTN), adhesion molecules (VCAM1, CD44) (Chen et al. 2022; Srivastava et al. 2015), and numerous proteases (MMPs) (Tao et al. 2021), while decreasing inhibitors like TIMP1 (Di Gregoli et al. 2016), a signature of plaque vulnerability. Calpain‐1 ablation reversed this destructive signature, decreasing collagen/matrix/adhesion molecules and proteases while increasing serpin inhibitors. This coordinated shift promotes a more stable plaque phenotype less prone to rupture. Parts of these findings were supported by the previous reports showing that sinigrin attenuates atherosclerosis potentially by inhibiting VCAM‐1 expression (Jang et al. 2017); corilagin ameliorates atherosclerosis by regulating MMP‐1, ‐2, and ‐9 expression in vitro and in vivo (Tao et al. 2021) and N‐acetylneuraminic acid and chondroitin sulfate modulate CD44 receptor for the efficient therapy of atherosclerosis (Chen et al. 2022).

Oxidative stress and mitochondrial dysfunction are increasingly recognized as major contributors to vascular inflammation and atherosclerosis progression (Jang et al. 2025). The present study revealed that calpain‐1 KO decreased pro‐oxidative enzymes (HPGDS, PAOX) (Ouyang et al. 2022) driving ROS and inflammatory eicosanoids while simultaneously increasing key antioxidant defenses (GSTM6) (Hu et al. 2024) and restoring mitochondrial regulators (PDHA1, CS) (Sun et al. 2025; Tang et al. 2024; Wang et al. 2022; Zhongxin et al. 2025). This reversed the ApoE KO‐induced elevation in mitochondrial ROS, impaired detoxification, and compromised energy metabolism (pyruvate oxidation, TCA cycle, ETC efficiency), crucial for maintaining cellular health in the atherosclerotic milieu.

An important finding of the present study is that calpain‐1 deletion not only attenuated vascular pathology but also significantly improved cardiac function. In the present study, 9.4 T small‐animal MRI was used to detect the heart structure and function, providing superior spatial and temporal resolution and enabling accurate and reproducible quantification of ventricular volumes and functional parameters in murine models (Chen et al. 2024). The results of cardiac MRI showed that ApoE KO mice developed ventricular dilation and systolic dysfunction, characterized by increased LVESV and LVEDV and reduced LVEF. These findings are consistent with previous reports indicating that chronic atherosclerosis and systemic inflammation can promote ventricular remodeling through increased vascular stiffness, impaired coronary perfusion, and endothelial dysfunction. Notably, calpain‐1 deletion significantly improved cardiac systolic function, as evidenced by increased LVEF, LVSV, and partial normalization of ventricular volumes. Importantly, these improvements occurred without significant changes in serum lipid or glucose levels, indicating that the cardioprotective effects of calpain‐1 deficiency are likely mediated through vascular‐cardiac coupling mechanisms rather than systemic metabolic regulation. Several mechanisms may contribute to this functional recovery. First, calpain‐1 ablation improved endothelial function, as demonstrated by increased eNOS activity and NO bioavailability. Enhanced endothelial NO signaling can reduce vascular resistance, improve coronary perfusion, and decrease ventricular afterload (Yu et al. 2018). Second, the proteomic analysis revealed normalization of cytoskeletal remodeling and ECM pathways, which may stabilize vascular structure and reduce inflammatory signaling that adversely affects cardiac function (Zhang, Feng, and Kong 2024). Third, restoration of mitochondrial metabolism and antioxidant defense may improve cellular energy homeostasis in both vascular and cardiac tissues (Liu et al. 2022). Collectively, these findings suggest that calpain‐1 may act as a molecular link between vascular pathology and cardiac dysfunction, and its inhibition may simultaneously mitigate atherosclerosis and cardiac remodeling.

Several limitations of the present study should be acknowledged. First, although DIA‐based proteomic profiling identified multiple molecular pathways potentially regulated by calpain‐1, additional functional validation of specific targets will be required to further confirm these findings. Therefore, the proteomic analysis in this study should be considered hypothesis‐generating, providing a basis for future mechanistic investigations. Second, while proteomic analysis provides a comprehensive overview of protein‐level alterations, transcriptomic approaches such as RNA‐seq were not performed, and future studies integrating multi‐omics analyses may provide deeper insights into the regulatory networks associated with calpain‐1. Third, although cardiac structure and function were evaluated using high‐resolution 9.4 T small‐animal MRI, blood pressure measurements were not included in the present study, which may further help to clarify the hemodynamic contribution to vascular–cardiac interactions. Finally, longer experimental durations may provide additional insight into the role of calpain‐1 in advanced plaque instability and progressive cardiac remodeling.

In conclusion, this study demonstrates that calpain‐1 contributes to atherosclerotic plaque development and cardiac dysfunction in ApoE‐deficient mice. Genetic deletion of calpain‐1 significantly reduces plaque burden and improves cardiac function through lipid‐independent mechanisms involving suppression of foam cell formation, restoration of endothelial function, stabilization of extracellular matrix remodeling, and normalization of mitochondrial metabolism. These findings highlight calpain‐1 as a promising therapeutic target that links vascular pathology with cardiac remodeling in atherosclerosis.

## Author Contributions

**Youming Shi:** conceptualization, methodology, software, investigation, formal analysis, supervision, funding acquisition, writing – original draft, writing – review and editing, project administration. **Havyarimana Juvenal:** conceptualization, methodology, investigation, validation, writing – original draft. **Yunqi Pan:** methodology, investigation. **Shuangyi Li:** methodology, investigation, formal analysis, visualization. **Wenjiao Gu:** methodology, investigation, validation. **Yujie Wei:** methodology, investigation, formal analysis. **Ying jiang:** methodology, investigation, formal analysis. **Junjie Shao:** methodology, investigation, validation. **Sijing Chen:** methodology, investigation, formal analysis. **Yunyang Zhao:** methodology, investigation, validation. **Ting Liu:** methodology, investigation. **Limei Shuai:** methodology, investigation. **Shining Zhang:** methodology, investigation, formal analysis. **Jixin Liu:** conceptualization, methodology, funding acquisition, formal analysis. **Xiaochun Zhou:** methodology, investigation, validation, supervision, project administration. **Futian Tang:** conceptualization, methodology, software, investigation, formal analysis, supervision, funding acquisition, writing – original draft, writing – review and editing, project administration.

## Funding

This work is supported by Fundamental Research Funds for the Central Universities of Lanzhou University (lzujbky‐2022‐sp08); Undergraduate Innovation and Entrepreneurship Training Program of Lanzhou University (20250050159); Grant of Gansu Provincial People's Hospital (20GSSy318); Major Science and Technology Project of Gansu Province (22ZD6FA050); Medical Innovation and Development Project of Lanzhou University (lzuyxcx‐2022‐154, lzuyxcx‐2022‐141); National Natural Science Foundation of China (81870329, 81960673); Project of Gansu Provincial Department of Education (2021jyjbgs‐02); Project of Gansu Provincial Development and Reform Commission (2020–2024); Natural Science Foundation of Gansu Province (21JR1RA135, 23JRRA1001); and Gansu Province Clinical Research Center for Digestive System Diseases (18JR2FA007).

## Ethics Statement

All experimental procedures were performed in accordance with NIH guidelines and approved by the Ethics Committee on Animal Study of Lanzhou University Second Hospital approved the protocol (No. D2022‐191).

## Conflicts of Interest

The authors declare no conflicts of interest.

## Data Availability

The data that support the findings of this study are available from the corresponding author upon reasonable request.
